# Searching for Evidence-Based Public Policy and Practice: Analysis of the Determinants of Personal/Public Adaptation and Mitigation Behavior against Particulate Matter by Focusing on the Roles of Risk Perception, Communication, and Attribution Factors

**DOI:** 10.3390/ijerph18020428

**Published:** 2021-01-07

**Authors:** Geunsik Kim, Seoyong Kim, Eunjung Hwang

**Affiliations:** 1 Institute of Government Studies, Korea University, Seoul 02841, Korea; kimgeunsik78@nate.com; 2 Department of Public Administration, Ajou University, Suwon 16499, Korea; 3 The Convergence Institute of Healthcare and Medical Science, Catholic Kwandong University, Incheons 22711, Korea; 2ejhwang@gmail.com

**Keywords:** particulate matter, attribution factor, response action, risk perception, risk communication, blame attribution

## Abstract

In recent years, awareness about the risk of particulate matter (PM) has increased both domestically and internationally; consequently, various policies have been implemented to reduce PM. Since citizens are both victim and cause of this risk, PM cannot be successfully decreased only through government policies. Therefore, citizens’ active participation is required to reduce PM and prevent its risks. However, a theoretical model about public behavior against PM has not been established. Therefore, we suggest the public behavior model about individuals’ response against PM, in which response actions are classified into four types based on the combinations of the public-personal domains and mitigation-adaptation actions: Personal or public adaptations, and personal or public mitigations. We analyze how risk perception, risk communication, blame attribution factors influence the four types of responses against PM. The analysis results reveal that the receiver’s ability, negative emotion, trust in government, and age influence personal mitigation behavior, personal adaptation, public mitigation, and public adaptation, respectively. As this study demonstrates the differences in the factors influencing each type of response actions against PM, evidence-based policy is needed that considers the differences in these influencing factors.

## 1. Introduction

Particulate matter (PM) is a small air pollutant that is invisible to the eye; “PM_2.5_” in particular is smaller than PM_10_ and more harmful [1]. According to a report by the Intergovernmental Panel on Climate Change [2,3], the use of fossil fuels primarily results in PM. PM_2.5_ is a serious pollutant worldwide from a human health perspective. Even with moderate levels of PM_2.5_, chronic exposure significantly increases the risk of heart disease and stroke, the leading causes of death in OECD countries [4]. The World Health Organization (WHO) [5] defined PM_2.5_ as the material that has the greatest negative impact on health among air pollutants. Worldwide, PM_2.5_ in the air ranks fifth among causes of death, after causes such as high blood pressure and smoking [6].

**Figure 1:**
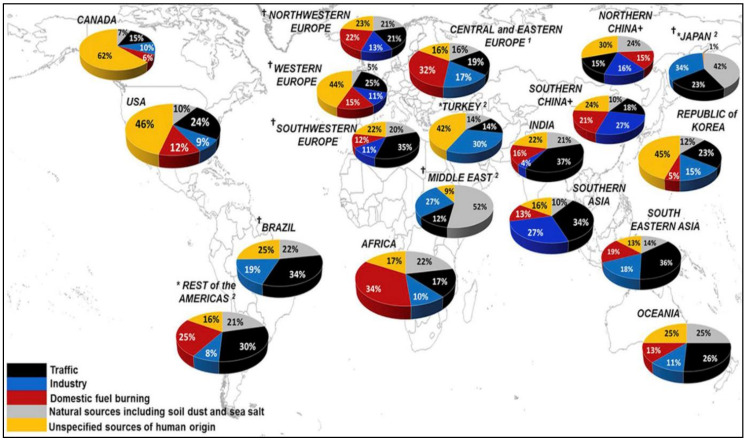
Population-weighted averages for relative source contributions to total PM2.5 in urban sites. *, † regions in which (*) unspecified sources of human origin and (†) domestic fuel burning sources have not been assessed. 1 Based only on one study including domestic fuel burning, and therefore only provides indicative results. 2 Based only on two studies, and therefore only provides indicative results. Source: Karagulian et al. [7].

Emissions from transport, industry, electricity generation, agriculture, and domestic (household) sources are the main contributors to PM. Karagulian et al. [7] estimated typical shares of the ambient sources of PM_2.5_ by country as shows in Figure 1. They showed that 25% of urban ambient PM_2.5_ was contributed by traffic, 15% by industrial activities including power generation, 20% from domestic fuel burning, 22% from unspecified sources of human origin, and 18% from natural dust and sea salt (p. 479). PMs from neighboring countries were another source of PM emissions. For instance, in the case of Korea, for a high concentration episode (24 February 2014), the contribution rate of neighboring countries was analyzed as 51.94% [8].

The risk of PM is characterized by complex cause and response patterns, uncertainty, and broad impacts [9]. Since PM risk involves new factors in the development of science and technology, it is increasingly generated from industrialization based on such science and technology development. Further, new technology has been developed to measure materials including PM that cannot be seen with the naked eye. The identification of these dangerous substances has increased the awareness of their dangers. As the awareness of PM risks has significantly increased both domestically and internationally, various public policies have been devised to reduce the negative health effects of PM. 

In Korea, the health risks from PM have become serious. Therefore, the weather forecast has become important, as it involves PM, with a significant influence on the public’s outdoor activities. The WHO warns that danger occurs if the daily average PM_10_ exceeds 50 µg/m^3^ and PM_2.5_ exceeds 25 µg/m^3^. In 2018, 122 days exceeded 25 µg/m^3^ in Seoul, Korea’s largest city [10]. The Korean government has promoted various comprehensive policies in response to PM since February 2019, when it enacted the Special Act on PM Reduction and Management (14 August 2018). For example, the government has notified the dangers of PM to the public by continuously reporting on its risks and implementing PM forecasting and warning systems. However, those government effort has a limit to induce behavioral changes, such as the public’s response actions to prevent the health risks from PM [11]. Since the public is both a victim and producer of PM risks [12], it is difficult for them to actively reduce PM and prevent its risks.

Public attitudes and reactions to PM is complex. For example, people could change their attitude and behavior according to their perceived attribution structure by which they find the cause of PM. According to Heider [13], people exhibit different behaviors according to both internal and external attributions. In other words, various actions or intentions toward policies to prevent PM can vary according to how individuals define the responsibility and blame for PM. However, empirical studies scarcely address the role of the blame attribution for PM.

Although several PM studies have been conducted in terms of natural science, social science research has only recently begun to attract attention to PM issues. Therefore, no clear theoretical model has been presented to explain the factors influencing human behavior related to PM. Although research on the perception of PM-related environmental risks has been primarily conducted in Korea and China, such studies are in early stages [14,15]. Since few empirical studies have addressed various behavioral factors’ influences and effects on PM, it is necessary to analyze the factors that influence individuals’ responses to PM empirically.

This study aimed to analyze how three factors such as perception, communication, and blame attribution, affect individuals’ actions in response to PM. We compare the determinants of four types of response actions: personal or public adaptations, and personal or public mitigations.

## 2. Theory and Hypotheses

### 2.1. Literature Review

Although PM has various definitions, it is generally described as involving fine particles of less than 10 µm, and is scientifically described as an aerosol. Cetin et al. [16] defined PM as fine solid or fluid particles in a gas that occurs due to natural causes or artificial activities, and is classified as either PM_10_, PM_2.5_, or PM_1.0_ according to particle size. Further, PM is used as a key indicator of the air pollution that occurs in the atmosphere through various human activities [17].

Most PM studies have focused on health effects from it. Specifically, highly concentrated PM can be dangerous because it adversely affects the environment as well as human health [18,19]. Further, the poor air quality caused by PM negatively affects psychological health, such as depression, tension, and irritability, and causes direct negative impacts on various organs, such as the lungs and respiratory tract [20,21]. These health risks have compelled the WHO [4] to designate PM as a first-class carcinogen. Moreover, PM has become a social problem domestically and globally due to the negative effects on both the air environment and human health that transcend national borders [22,23]. Many PM studies are being actively conducted that focus on its environmental impacts [24,25,26]. 

Risk studies from social science have focused on the impact of risk perception and communication on individuals’ action against PM. Research on risk perception focuses on the effects of perceived risk, benefits, negative emotions, knowledge, and trust on response actions against PM, while studies on risk communication focus on credibility in the information source, information itself, and receiver’s status. In risk perception studies, public’s actual risk perception depends on subjective assessments rather than objective ones. Further, the public may follow the appropriate preventive actions differently, because they may express different views toward the same risk. The public tend to perform subjective risk assessments through intuition [27]. Therefore, Slovic et al. [27] argued that individuals’ perception depends on emotions and intuition when judging risk targets, and that these feelings and intuitions are based on empirical experience.

In the other hand, risk communication research focuses on the structural context rather than individual actors’ risk perceptions. The process of generating, transmitting, and interpreting information is an important factor in judging risk. As a result, the interactions between the senders, media, and receivers of the information influence the individuals’ perceptions of risk. Rather than micro-level individual actions, the interactions between actors, between actors and media in the macro-societal context play a role in spreading or reducing social risk perceptions.

Therefore, the next section introduces our hypothesis regarding how each specific variable of risk perception and communication factor affects the actions in response to PM. Our hypothesis also addresses the effects of blame attribution factors that have been overlooked in previous studies on the response actions against PM.

### 2.2. Risk Perception Factor

#### 2.2.1. Perceived Benefit

The health belief model presents the theoretical concept and role of perceived benefits, which refer to the degree to which one believes that taking a particular action could largely reduce health risks or threats [28]. Further, Chung [29] found that the perceived benefits have a statistically significant positive effect on including response action to decrease PM. Kim and Park [30] also demonstrate that the perceived benefit from addressing the problem of PM positively impacts the actions in response to PM. Kim and Kim [31] observe the positive role of perceived benefits, in which the greater the benefit through the reduction of fire, the greater the intention to respond to climate change. Similarly, as the benefits from eliminating PM dust increase, the response behavior would increase. Thus, we propose the following Hypothesis 1:

**Hypothesis** **1** **(H1).** *The perceived benefit will positively influence actions against PM*.

#### 2.2.2. Perceived Risk

The risk from PM has significant negative health impacts; the elderly in particular are more likely to face deteriorating health effects from PM than other groups are [32]. The PM risk is particularly problematic given the degree of damage in terms of severity, routine/persistence, period for problem-solving, invisibility in terms of sensory evaluation, and historical ambiguity of causes and results [33]. Previous studies on the risk of PM in Korea revealed that risk perceptions of PM have a relatively greater influence on the behavioral intention toward prevention than other factors [11]. Thus, we propose the following Hypothesis 2:

**Hypothesis** **2** **(H2).** *Perceived risk positively influences actions against PM*.

#### 2.2.3. Knowledge

If people have a complete knowledge about PM, risk perceptions about it will decrease among them [34]. Generally, knowledge is recognized as an important factor in determining the risk perception. It usually relates to science and technology. Therefore, a lack of knowledge about modern science and technology will induce the public’s skepticism toward these fields [35]. This will ultimately induce the public’s distrust toward scientific and technological artifacts. Moreover, if the public may have an insufficient understanding of more complex sciences and technologies, such as nuclear power, people may consequently regard them as dangerous [36]. Knowledge drives citizens’ cooperation and participation in public policies. For example, Shi et al. [37] discovered that the knowledge of climate change significantly correlates with concerns about it and the intention toward actions to mitigate it. As knowledge positively influences the public’s acceptance of policies related to risky climate change, we propose the following Hypothesis 3:

**Hypothesis** **3** **(H3).** *More knowledge will positively influence actions to mitigate PM*.

#### 2.2.4. Negative Emotion

Recent risk studies have focused on not only cognitive but also emotional factors in risk judgment. Loewenstein et al. [38] demonstrated that the public tend to prioritize emotional judgments over cognitive in the risk-perception process. Slovic et al. [39] showed the role of emotion as affective heuristic processing, in which emotion is significant in making decisions or judgments. Since emotional judgments occur more rapidly than cognitive judgments in complex and uncertain situations, this helps rapid decision-making [40]. Further, Loewenstein et al. [38] argued that aside from cognitive judgment, emotions independently influence decision-making and behavior in risk situations. In risk communications, anger and anxiety are regarded as the emotional sides of risk perceptions [27]. In dangerous situations, emotions closely relate to behavioral intentions. Therefore, emotions such as anxiety, worry, and fear are triggering behaviors to prevent potential risks [41,42,43,44]. As a result, we propose the following Hypothesis 4:

**Hypothesis** **4** **(H4).** *Negative emotions will positively influence actions to mitigate PM*.

#### 2.2.5. Trust

Previous studies observe that trust is an important influence in risk judgment. Visschers et al. [45] divided trust into social and institutional trust. Social trust depends on beneficial outcomes expected from another, while institutional trust does whether institutions provide risk-related information in managing risk. Trust itself is people’s level of confidence toward individuals, organizations, and institutions, which usually concern with risk objects [46]. As the public may have insufficient knowledge regarding the dangers of complex sciences and technologies, they may exhibit a high tendency to rely on trustworthy actors such as experts, governments, and public organizations. Trust in empirical studies is generally measured through institutional trust [45,47,48,49]. We propose the following Hypothesis 5:

**Hypothesis** **5** **(H5).** *A greater trust in government benefit will positively influence actions to mitigate PM*.

### 2.3. Risk Communication Factor

#### 2.3.1. Source Credibility

Risk communication studies have examined how various information sources have influenced individuals’ risk judgments [50]. An sources credibility is audience’s belief regarding the information source; this depends on the audience’s subjective evaluation, not the information source itself [51,52,53]. Credibility in information sources can involve interpersonal trust, which depends on the presence or absence of a particular perceived characteristic for a specific information source [50].

Various sources of information provide PM information, such as family, colleagues, friends, experts, the media, and the government. However, the general public mainly received information from government agencies’ websites or Internet applications, and tend to evaluate these sources as accurate and expertise. The government was generally recognized as a primary information source [54]. Further, Szykman et al. [55] found that people’s attitudes toward public messages related to drunk driving could vary depending on their source. Individuals more positively evaluated public interest campaign messages sponsored from non-profit organizations than for-profit ones because the former has a higher persuasive effect than the latter. Thus, we propose the following Hypothesis 6:

**Hypothesis** **6** **(H6).** *A source’s credibility will positively influence actions to mitigate PM*.

#### 2.3.2. Quality/Quantity of Information

The quantity and quality of information can be considered important factors influencing risk perceptions because they decrease the uncertainty of risk. As PM is a topic involving issues that people face daily, the information’s impact on the risk perception of PM may differ depending on the frequency of exposure to it. Ford [56] demonstrated that excessive exposure to dangerous information strengthens risk perceptions, and reduces safety-related effects.

The quality of information is the degree to which the public subjectively perceives how persuasive powers are embedded into the arguments in a message [57]. The quality of information contributes to logical, accurate judgments; the greater the quality of information present in a message, the higher the persuasive effect [58]. Moreover, Lee et al. [59] demonstrated that message intensity as a qualitative characteristic of information promotes the change in risky behaviors, such as smoking. This implies that the greater the quality of risk-related information, the stronger the persuasive power, which will ultimately lead to changes in attitude and behaviors. Thus, we propose the following Hypothesis 7:

**Hypothesis** **7** **(H7).** *A greater quality and quantity of information will positively influence actions to mitigate PM*.

#### 2.3.3. Receiver’s Ability

The information receiver’s ability refers to their degrees of cognitive information processing, which affects the information-processing mode as well as final decisions [60]. The greater the cognitive ability, the more systematic the information processing whereas the lower the cognitive ability, the higher the possibility of heuristic-based information processing [61]. According to Todorov et al. [62], systematic information processing relies on personal resources and motivation because it requires the extensive processing of persuasive information. However, heuristic processing focuses only on the information leading to the use of simple decision-making rules, and thus, it is less restricted by motivation and cognitive resources. Therefore, Chaiken and Maheswaran [63] argued that when individual receivers have a lower ability, they are more influenced by heuristic cues, such as the length of the message or other people’s opinions, than by the message’s quality. On the other hand, those who have a higher information-processing ability believe that they can sufficiently overcome external risk, and will tend to evaluate the current risk as relatively low. Matthews and Moran’s [64] comparative study of perceived driving ability and risk perceptions by age indicated that young people valued their driving ability as relatively high, although they underestimated the possibility of accidents. Thus, we propose the following Hypothesis 8:

**Hypothesis** **8** **(H8).** *A receiver’s greater information-processing ability will positively influence actions to mitigate PM*.

### 2.4. Attribution of Blame and Responsibility

Attribution refers to the process of inferring the cause of an event [8]. According to the attribution theory, inference about the cause or intention of an event or action will change people’s attitudes or opinions according to attribution type and mode [65,66]. Further, Heider [67] argued that people give meaning to their environments while searching for causes for various social events. The difference in meanings depends on whether an event’s cause is internal or external. Rotter [68] classified an event as an internal control attribution if it was caused by a controllable internal factor, while it would be attributed to external control if it was caused by an uncontrollable external environmental factor. Niederdeppe et al. [69] proposed that attribution tends to appear as a problem of accountability, in that the subject that caused a specific event is responsible for solving the problem. Weiner [70,71] explained that the idea of what causes social problems (the cause attribution) influences who is responsible for the problem (the responsibility attribution). These attributions affect individual actions as well as government policies for problem solving. The core of attribution theory is accountability and the emotional reactions which motivate to accomplish the specific actions and to induce persuasive mechanisms to search for alternative solutions for problems. Finally, attribution is a cue that acts as a driving force for problem-solving [72].

Both internal and external attribution can affect individual behavior, but in different ways. Rothman et al. [73] analyzed the correlation between the responsibility attribution and persuasion effects through persuasion messages for breast cancer screening. They showed that women who received an internal attribution message emphasizing their own responsibilities had more positive attitudes and behavioral changes toward the examination than those who received an external attribution message emphasizing the doctor’s responsibility. Kang and Kim [8] demonstrated the effect of the attribution of PM generation and the information source’s reliability on the intention to respond to PM. They discovered that the attribution frame directly affects the intention to respond to PM. The greater the internal attribution than the external attribution, the greater the intentions toward both preventive actions and policy participation. Similarly, other previous studies reveal that internal attribution messages cause more positive attitudes and behavioral changes than external attribution messages [73,74]. Thus, we propose the following Hypothesis 9:

**Hypothesis** **9** **(H9).** *An internal attribution increases actions to mitigate PM, while an external attribution decreases them*.

### 2.5. Adaptation and Mitigation Action

As the risk of PM has become both socially important and serious, individuals’ efforts to reduce PM—as well as national-level public policies—have become increasingly important [75]. To promote the implementation of national-level actions required to reduce PM, a positive communication strategy is needed to awaken individuals’ involvement in related policies and mitigation measures. For example, public communications could involve delivering news through newspaper media about weather forecasts for PM, its causes, and danger levels, and providing behavioral information to the public. At the governmental level in Korea, the Ministry of Environment has pursued a policy to provide relevant information continuously through the production and distribution of information materials on personal guidelines of conduct when PM warnings occur.

Responses against the risk of PM can be largely divided into two types: mitigation and adaptation [76]. In terms of science and technology, mitigation measures include reducing the root cause of the risk associated with PM [77], while adaptation measures involve controlling the damage caused or incurred by PM, by using natural or artificial systems [2]. Such adaptation and mitigation measures have become very important for national-level environmental policy related with PM. Moreover, these adaptations and mitigations can apply to individual-level attitudes and behaviors.

As the public is both a victim and producer of environmental risks, their efforts are as important as responding to public policies to prevent and reduce PM at the national level. At the individual level, mitigation actions to reduce the risk of PM include the use of public transportation to reduce automobile emissions, the use of kitchen utensils that generate less harmful wastes, and actions to reduce the sources of PM. Alternatively, adaptation actions include wearing a mask to prevent damage from PM, and such preventive actions as refraining from venturing outside at times of high PM density. However, given the nature of adaptation and mitigation, the former is a passive response to a given situation, while the latter implies the active action of changing the context. These difference characteristics inevitably relate to differences in the factors influencing the two behaviors.

This study divided adaptation and mitigation actions into personal and public dimensions in consideration of the sphere in which those actions occur. Actions in personal sphere can occur immediately within the range of personal control whereas actions in public spaces should be performed in cooperation with actors as well as members of the community. Both personal and public contextual domain can be simultaneously considered in doing mitigation and adaptation actions in dealing with PM. Mitigation is focused on minimizing the negative impact of the PM and addressing the cause of it, while the adaptation is on making good use of the opportunities offered in negative situations. Two (personal/public sphere) by two (mitigation/adaption) matrix can be divided into four response action against PM, as illustrated in Table 1.

The determinants of response action against PM depends on both contextual domains in which the action takes place and the different types of action by which people take. Therefore, we propose the following Hypothesis 10:

**Hypothesis** **10** **(H10).** *Four response actions show different determinant structure*.

**Figure 2:**
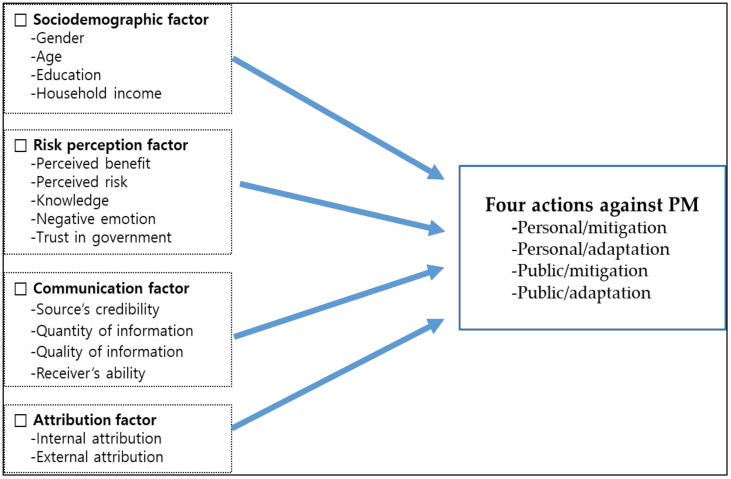
Research model.

Figure 2 illustrates this study’s research model as based on the previously mentioned theoretical hypotheses.

## 3. Sample and Measures

This study used survey data (*N* = 1020) collected through an online survey. Researchers were in charge of designing the survey questions; Korea Research, a specialized public opinion survey, was responsible for surveying respondents. The survey was conducted for seven days (12 to 18 January 2019). We used the online panel managed by Korea Research, which consisted of 350,000 people. 16,736 e-mails were sent, 2289 e-mails were opened. Finally, 1843 respondents participated in the survey. After excluding incomplete responses, we were left with 1020 respondents. Of the survey devices used by the respondents, 665 were personal computers and 355 were mobile devices. A quota-sampling method was used to match the proportion of respondents to the distribution ratio of Korea’s general population, and this considered region, gender, and age groups. Table 2 displays the distribution of respondents according to demographic variables.

Of the 1020 samples used in this study, 486 (47.6%) were male and 534 (52.4%) were female, indicating a slightly higher proportion of females. Respondents were also divided into two groups according to educational background, with 568 (55.7%) and 452 (44.3%) having a high school degree and higher, respectively. By age, the groups consisted of 161 people (15.8%) in their 20s, 167 in their 30s (16.4%), 207 in their 40s (20.3%), 207 in their 50s (20.3%), and 278 people (27.3%) in their 60s or older. The respondents had a smaller proportion of those in their 20s and 30s, while a higher proportion of those in their 60s and older. In terms of household income, 389 people (38.1%) earned three million won or less, 370 (36.3%) did between three and five million won, and 261 (25.6%) did more than five million won.

This study selected three factors as independent variables affecting the response actions against the blame, risk perception, and risk communication factors. The attribution factors were classified as either domestic or foreign. The former refers to attributing the occurrence and cause of PM to domestic companies or governments, and the latter to China or other countries. Risk perception factors were classified into the perceived benefits from solving the PM problem, the perceived risk of diseases caused by PM, knowledge of PM, negative emotions about PM, and trust in government’s public policy about PM. The risk communication factors included credibility in the information source, the quantity and quality of PM information, and the receivers’ ability. Two or more items were used to measure the detailed variables for the three factors, as displayed in Table 3. A five-point Likert scale was used to gauge the response to each question, with five possible options: 1, or “I do not agree at all”; 2, or “I disagree”; 3, or “Neutral”; 4, or “I agree”; and 5, or “I strongly agree.”

## 4. Analysis and Findings

### 4.1. Descriptive Analysis

We conducted a basic statistical analysis to not only examine the extent of differences that exist in the level of actions in responding to PM but also determine the influencing factors according to demographic characteristics.

From Table 4, among the four types of response actions, the average personal adaptation response action was the highest at 3.71, indicating that prevention response action would easily occur at the individual level. Personal adapted behaviors are easy to practice because individuals can control them. Next, the average of the personal-mitigation response actions was 3.45. While adaptation and mitigation often occur more in the sphere of the personal domain than the public one, the average public-adaptation response action was 3.29, and the average of public-mitigation response actions was the lowest, at 2.93. Public mitigation actions include donating to environmental groups for reducing PM, paying their necessary expenses, and intending to join groups that act against PM.

In examining the differences in actions according to demographics, first, the statistically significant differences in response action by gender were found only in the personal-adaptation response. In other words, women were aware of personal-adaptation actions, such as refraining from venturing outside and using masks, and expressed a greater willingness to manage air quality, more than men do. These results can imply that women understand better the risk of PM possibly threatening their health or others’ one, including that of their children.

Regarding educational level, the respondents with a high school education had a greater awareness of personal adapted response actions than university graduates did, such as refraining from venturing outside and using masks, and were more willing to manage air quality.

Regarding income level, the results revealed significant differences in public-level response actions. Both public-adaptation response actions, such as donating to and joining environmental organizations, and public-mitigation response actions, such as supporting the decreased use of diesel vehicles, prohibiting older vehicles from cities, and closing coal-fired power plants, occurred more often in the higher-income groups. This suggests that public actions are supported by various costs and resources.

By age group, statistically significant results were observed for all actions. Overall, the four actions were more often exhibited among those in their 50s and 60s than in their 20s to 40s. This result implies that the older the age, the poorer the health status, and the greater the health damage caused by PM; thus, more active behaviors occur.

As noted in Table 5, we examine the correlation coefficients of the relationship between variables in the demographic, risk perception, communication, attribution and the four response actions.

First, where we observe demographic factors; regarding gender, females had a positive relationship (+) with the personal-adaptation response. Thus, women are more aware of personal adaptation responses to PM than men are. Age exhibited a positive relationship (+) with personal mitigation, personal adaptation, and public adaptation actions; therefore, as age increases, the three actions against PM increase. As previously mentioned, this result seems to be induced by elderly’s health concerns. The educational level demonstrated a positive relationship (+) with personal mitigation and adaptation actions; subsequently, the higher the educational level, the more personal mitigation and adaptation responses to PM occurred. The enlightening effect of education seemingly induces a response to PM. However, higher education had no statistically significant correlation with the two public behavioral responses. Additionally, household income did not exhibit a statistically significant correlation with the four actions against PM, which suggests that the responses to PM are not simply a matter of economic wealth.

Next, in observing risk perception factors, it was found that perceived benefit, perceived risk, subjective knowledge, and negative emotion positively correlated with all four actions against PM. Given the high correlation between knowledge variables and response actions, this raises the need for knowledge accumulation and diffusion. Generally, risk perception studies show a positive correlation between a perceived benefit, knowledge and the four actions against PM. However, perceived risk and negative emotions positively relate to actions, which is a contrasting with existing risk perception studies. For example, Tanaka [78], Flynn et al. [79], and Sjӧberg [80] (2004) demonstrated that perceived risk and negative emotions negatively correlate with such factors as the acceptance of risk targets. This difference seems to depend on the negative nature of the dependent variable, which generally involves the acceptance of risky objects in previous studies; however, our study’s predicted variables are more positive objects. Finally, trust positively correlates with three actions against PM, excluding personal adaptation actions.

Examining the correlation between risk communication factors and actions against PM revealed that the source’s credibility, quality of information, and receiver’s ability positively correlated with all four actions against PM. The positive correlation exists between the quantity of information and three actions against PM, except personal adaptation. The correlation coefficients between the receiver’s ability and response actions behavior are high, suggesting that personal competency can promote these behaviors.

Regarding the correlation between the attribution factors for PM and the four types of action against PM, internal attribution positively correlated with the three actions against PMs, except for the public mitigation action. Moreover, external attributions positively correlate only with personal adaptation responses, which suggests that internal attribution induces greater response actions to PM than external attribution does.

### 4.2. Regression Analysis

#### 4.2.1. Personal Mitigation Action

Table 6 displays the results from the regression analysis, which was performed to analyze the determinants of the four response behaviors. Among demographic variables, age had statistically significant and positive effects on response action, the higher the age, the greater the personal mitigation action.

Regarding the risk perception factor, the perceived benefits and negative emotions positively influenced the personal mitigation response; therefore, the greater the perception of benefits from solving the PM problem, the more positively this affects the personal mitigation action. Additionally, the negative emotion toward PM causes the personal mitigation action to trend in a positive direction. These results are consistent with previous research findings, in that such negative emotions as anxiety and fear in dangerous situations positively influence behaviors to prevent potential future risks [42,43,44,45]. Regarding communication factors, the receiver’s ability positively affected personal mitigation actions. This indicates that programs to improve the receiver’s ability are necessary to increase actions and policies to mitigate PM. Attribution factors did not statistically affect response behavior. In observing the standardized regression coefficient values, the receiver’s ability had the largest value, followed by the perceived benefit. The finding that the receiver’s ability had the greatest degree of influence on personal mitigation actions, indicating the need for policies to improve the receiver’s ability. Moreover, it is necessary to emphasize the consequential benefits of responding to PM when communicating with the public.

#### 4.2.2. Personal Adaptation Action

Among demographic factors, gender, age, and educational level had a statistically significant influence. Men exhibited fewer personal adaptation actions than women did. Personal adaptation actions increased with age and higher education. Regarding risk perception factors, the perceived benefit, perceived risk, and negative emotions influenced personal adaptation action. Of the three risk factors, the standardized regression coefficient for negative emotions was the largest. This suggests that personal adaptation behavior can be induced by appealing to negative emotional elements rather than emphasizing rational aspects. In terms of communication factors, the source’s credibility and the receiver’s ability positively affected personal adaptation actions; therefore, increasing trust in the government and organizations that provide PM information is critical in inducing actions against PM.

Among the attribution factors, it was found that the greater the external attribution, the higher the personal adaptation action. In other words, the more people who believe that the attribution to the cause of PM is not a domestic factor, but originates in such foreign countries as China, the more actively they will respond through personal adaptations. This can be interpreted to indicate that people tend to personally adapt as much as possible when they perceive that it is difficult to solve problems with PM. Such difficulties are aggravated when PM occurs abroad, such as in China. These results confirm findings by Rothman et al. [73] in that the internal attribution message positively affects health behavioral intention, while the external attribution message causes negative behavioral changes. 

#### 4.2.3. Public Mitigation Action

Among the demographic factors, age and education level had a statistically significant effect. Moreover, negative emotions and trust in policy influenced public mitigation action in the risk perception factor. The greater the negative emotion, the greater the public mitigation action. Therefore, negative emotions toward PM tend to increase public mitigation actions. Trust in the government’s policies was found to positively affect public mitigation actions; the relative influence of trust was the largest among all risk factors. This demonstrates that trust in the government’s PM policy is a critical factor in responding to PM in the public domain but not in the personal domain. Trust in government was not statistically significant in inducing personal action but was significant in inducing public action. Regarding communication factors, the receiver’s ability positively affected public mitigation actions. Specifically, as this ability had the second-largest degree of relative influence on public mitigation actions, it can be posited that policy measures must improve such abilities. Both public and personal mitigation actions require voluntary participation; therefore, voluntary participation must be emphasized when improving recipients’ ability to address PM and increase trust in PM policies.

Among the attribution factors, the higher the internal attribution, the lower the public mitigation action. These results are contrasting with findings from Kang and Kim [11], in which PM actions are influenced more by internal than external attribution, given the greater the intention to act toward prevention and participate in policy. Comparing the magnitude of the relative influence on public mitigation actions reveals the following order: trust in government > the receiver’s ability > negative emotions > educational level > internal attribution > age.

#### 4.2.4. Public Adaptation Action

Among the demographic factors, age had statistically significant effects; the higher the age, the higher the public adaptation action. Regarding the risk perception factors, negative emotions and trust influenced public adaptation action. As negative emotions affect all four response actions, policy programs must focus on invoking the public’s emotions. Additionally, as trust in government affects public mitigation and adaptation, policymakers must consider how to increase trust in the government to induce response behavior in the public domain. In terms of communication factors, information sources’ credibility positively affected public adaptation actions. Simultaneously, negative emotions, trust in government, and credibility in source are significant predictors, which suggests that people’s perceptions as well as methods of communication should be considered when inducing response behaviors.

Attribution factors were found to have statistically significant effects on public adaptation actions for both internal and external attributions. The higher the internal attribution, the higher the public adaptation action, while the higher the external attribution, the lower the public adaptation action; however, this indicates contrasting effect. These conflicting results can be interpreted as reflecting the relative possibility of control. Generally, as problems are controlled in Korea, it is possible to induce response actions at the personal and policy levels. However, when the problem is caused outside the country, it is often impossible to control it, and thus, the response action is bound to decrease. This is consistent with previous studies demonstrating that internal attribution messages create more positive attitudes and behavioral changes than external attribution messages [72,73,74].

#### 4.2.5. Comparing the Determinants for Four Actions

A comparison of the four response behaviors while considering their similarities and differences reveals the following results. First, the three variables with the greatest positive impact on all four response behaviors are age, negative emotions, and the receiver’s ability. The perceived benefit variable has the greatest impact on personal action, while the trust variable strongly affects public behavior.

Second, when observing the variable’s degree of influence, personal mitigation was explained the largest by the receivers’ ability, personal adaptation by negative emotions, public mitigation by trust, and public adaptation by age. This difference in explanatory power suggests that the variables of emphasis can differ according to the resulting response behavior.

Third, some variables’ direction of influence may vary based on the dependent variable, which is the case with attribution. For example, intrinsic attribution reduces public mitigation but induces public adaptive behavior. These results may reflect differences in the characteristics of mitigation and adaptation; the former is an area beyond personal control, but the latter is possible to control by individuals.

Fourth, when observing the model’s overall explanatory power, personal adaptation (37.5%) exhibited the greatest explanatory power, followed by public mitigation (20.3%), public adaptation (17.4%), and personal relaxation (15.3%).

## 5. Summary of Findings

This study aimed to analyze the determinants of actions to mitigate PM. To this end, four such actions were derived by categorizing mitigation and adaptation at the personal and public domains. Additionally, four factors were selected as the determinants of responses to PM: demographic, risk perception, communication, and attribution factors.

The descriptive analysis indicated that the personal adaptive action scored the highest, with an average of 3.71; followed by personal mitigation (3.45), public adaptation (3.29), and public mitigation action (2.93). This implies that Korean citizens more highly value such as personal response actions as the use of masks and refraining from venturing outside than such public actions as support for policy changes.

A regression analysis was conducted to analyze determinants’ influence on the response to PM, with the following results. First, the receiver’s ability had the greatest influence on personal mitigation actions, followed by the perceived benefit, negative emotions, and age. Therefore, policy measures must be prepared to enhance individuals’ capabilities and ultimately promote personal mitigation actions. Moreover, the perceived benefit and negative emotions both positively affect personal mitigation. Second, in the case of personal adaptation actions, negative emotions had the greatest influence, followed by the perceived benefit, risk perceptions, the receiver’s ability, gender, the source’s credibility, age, educational level, and external attribution. Except for gender, all variables have a positive impact on personal adaptation. Third, in the case of public mitigation action, trust in government had the greatest influence on public mitigation action, followed by the receiver’s ability, negative emotions, higher education level, internal attribution, and age. Except for internal attribution, all variables have a positive impact on actions. The higher the propensity for internal attribution, the lower the public mitigation action. Fourth, in the case of public adaptation mitigation action, age had the greatest influence on public adaptation action, followed by negative feelings, trust in government, source credibility, perceived benefits, and external and internal attributions.

Table 7 displays the summary of the results from the regression analysis.

## 6. Discussion and Implications

This study provides theoretical implications, in that we discover a meaningful dimension of response actions by categorizing four groups of existing actions. To consider four actions instead of a single action, we include both public/personal actions and mitigation/adaptation ones. This classification of response behaviors enables a more elaborate approach in which public policy measures differ by action types when implementing policies.

Second, it is meaningful to observe that various determinants—such as communication, risk, and attribution factors—affect the responses to PM. Risk perception and communication and attribution factors affect all four behaviors; these results suggest that various factors, and not a single factor, should be simultaneously considered when inducing specific behaviors against PM.

Third, regarding the variables’ degrees of influence, the recipient’s ability, negative emotions, trust, and age showed the most explanation power for personal mitigation, personal adaptation, public mitigation, and public adaptation, respectively. This difference in explanatory power suggests that the variables of emphasis should differ according to the resulting behavior types.

Fourth, more effective variables should be considered when inducing changes in personal and public behavior. For example, trust in government should be increased when inducing public action, and perceived benefits should be emphasized when inducing personal action.

## 7. Limitations and Future Research

The validity and reliability of the types of response actions may be weak because this work is the first attempt to categorize them. As this study is nearly the first which theorized the actions against particulate matter, theoretical bases on four actions to mitigate PM have not been fully established yet. Further research on behavioral studies on PM will improve the study. In addition, in respect to the research model, this study assumes that the health or concern about health is only related to age. Considering other long term health conditions and disability, the model would be more rigorous. As the measurement items in response to personal mitigation behavior may be unreliable, this requires to develop more appropriate measurement items. Finally, in terms of the model’s explanatory power, the personal adaptation action is high at 37.5%, but the remaining models exhibit low explanatory power. In particular, this study overlooks not only a lot of perception and communication [81,82,83], but also structure [84,85,86,87,88,89,90] and cultural value factors [91,92,93,94,95,96,97]. This requires more a relevant variable exploration and model development in the future.

## Figures and Tables

**Table 1:** Typology of response actions against particulate matter (PM).

		Response Action	
		Mitigation	Adaptation
**Dimension**	**Personal** **sphere**	Using public transportation Purchasing eco-friendly vehicles	Refraining from venturing outside Wearing a mask when outside Actively managing air quality
	**Public sphere**	Donating to environmental organizations Signing PM-reducing organizations Paying necessary PM expenses	Supporting the prohibition of older vehicles from entering the city Supporting the reduction of coal-fired power plants Supporting the reduction of diesel vehicles

**Table 2:** Sample characteristics.

Variable	Division	Frequency (%)	Variable	Division	Frequency (%)
Gender	Male	486 (47.6)	Household Income	<3.0 MW	389 (38.1)
				3.1 MW–5.0 MW	370 (36.3)
	Female	534 (52.4)		5.1 MW <	261 (25.6)
			Age	19–29	161 (15.8)
Education	Under high school	568 (55.7)		30–39	167 (16.4)
				40–49	207 (20.3)
	College or higher	452 (44.3)		50–59	207 (20.3)
				60+	278 (27.3)

Note: The MW abbreviation denotes million won.

**Table 3:** Concepts, measurement items, and the scale’s reliability.

Factor	Concept	Measurement Items	Reliability
Demographic factors	Gender	- Male (1), Female (2)	**-**
	Age	- How old are you? Age: ()	**-**
	Education	- Please tell me your final educational level. 1. No school, 2. Elementary school graduate, 3. Junior high school graduate, 4. High school graduate, 5. University student, 6. University graduate, 7. Graduate school student, 8. Graduate school graduate or higher	**-**
	Household income	- What is the average monthly gross income for your household? Please include all household members’ income, including yourself. Monthly: () in 10,000 won	**-**
Risk perception factors	Perceived benefit	- Solving the problem with PM will greatly benefit our society. - When PM is resolved, our society will significantly develop.	0.781
	Perceived risk	- Diseases caused by PM have serious consequences. - Diseases caused by PM will greatly affect my life.	0.844
	Knowledge	- I know more about PM than other people do. - I can explain PM-related policies or issues to others.	0.800
	Negative emotion	- PM evokes fear to me. - PM makes me nervous. - I get annoyed when I come across information about PM. - I am anxious when checking information on PM. - I am worried when I see information about PM.	0.831
	Trust in government	- I am satisfied with the government’s PM policies. - I trust in the government’s PM policies. - The government seems to be trying to communicate with the public on the PM issue. - I was relieved to see the government’s efforts to reduce PM.	0.910
Communication factors	Source credibility	- How much do you trust the fine dust-related information provided by next subjects? (1) The government (2) PM-related organizations	0.723
	Quantity of information	- The government provides sufficient PM information. - I am satisfied with the amount of PM information provided by the government.	0.846
	Quality of information	- The PM information provided by the government is realistic and vivid. - The PM information provided by the government is easy to obtain. - The government quickly provides PM information. - The PM information provided by the government is what I need. - I am satisfied with the quality (level) of PM information provided by the government.	0.897
	Receiver’s ability	- I know how to get more PM information. - I can distinguish between truth and fiction by reviewing PM information.	0.763
Attribution factors	Internal attribution	- PM is generated by private companies rather than individuals and households. - Private companies, rather than individuals, are responsible for solving the problems with PM. - The government is more responsible than individuals are for the generation of PM. - The government should be responsible for solving the problems with PM, rather than individuals.	0.855
	External attribution	- PM is caused by air pollutants introduced from China, rather than domestic issues. - The main responsibility for solving PM lies with China rather than Korea. - PM occurs more often abroad rather than domestically. - Countries other than Korea are responsible for resolving occurrences of PM.	0.881
Action	Personal/ mitigation	- I will use public transportation instead of driving my own car to reduce PM. - I have purchased, or am willing to purchase, an eco-friendly (e.g., electric) car to reduce PM.	0.372
	Personal/ adaptation	- I will refrain from venturing outside on days with a high PM concentration. - I will use a mask when outside on days with a high PM concentration. - I am willing to manage indoor air quality actively through such methods as ventilation and indoor water cleaning.	0.821
	Public/ mitigation	- I am willing to donate to environmental organizations to reduce PM. - I am willing to pay for the costs needed to reduce the risk of PM. - I am willing to join an organization that works to reduce PM.	0.792
	Public/ adaptation	- Even if it adversely affects economic growth, the use of diesel vehicles should be reduced as soon as possible. - Even if it inconveniences me, older vehicles should be prohibited from entering the city. - Even if electricity rates increase, coal-fired power plants must be closed.	0.606

**Table 4:** Comparison analysis of the differences in mean response actions against PM.

Variable	Division	Personal/Mitigation		Personal/Adaptation		Public/Mitigation		Public/Adaptation	
		Mean	F-Value	Mean	F-Value	Mean	F-Value	Mean	F-Value
All		3.45		3.71		2.93		3.29	
Gender	Male	3.45	0.037	**3.56**	41.101 ***	2.96	1.013	3.28	0.093
	Female	3.46		**3.85**		2.91		3.30	
Education	Less than high school	3.42	2.759	**3.65**	8.650 **	2.90	2.659	3.30	0.510
	College or higher	3.49		**3.79**		2.97		3.27	
Household Income	>3 MW	3.39	2.324	3.66	2.629	**2.83**	8.521 ***	**3.23**	3.180 *
	3.1–5 MW	3.47		3.71		**2.93**		**3.31**	
	51 MW+	3.51		3.79		**3.08**		**3.36**	
Age	19–29	**3.37**	3.463 **	**3.61**	4.271 **	**2.76**	3.028 *	**2.97**	15.205 ***
	30–39	**3.35**		**3.68**		**2.93**		**3.18**	
	40–49	**3.39**		**3.63**		**2.96**		**3.30**	
	50–59	**3.55**		**3.72**		**3.03**		**3.42**	
	60+	**3.53**		**3.85**		**2.93**		**3.44**	

Note: * *p* < 0.05; ** *p* < 0.01; and *** *p* < 0.001.; Bold figures mean statistically significant case.

**Table 5:** Simple Pearson correlations.

Factor		1	2	3	4	5	6	7	8	9	10	11	12	13	14	15	16	17	18
Demographic	1. Gender (female)	1																	
	2. Age	−0.016	1																
	3. Education	−0.048	−0.103 ***	1															
	4. Household income	−0.039	−0.075 **	0.129 ***	1														
Risk perception	5. Perceived benefits	0.086 ***	−0.035	0.037	0.050	1													
	6. Perceived risks	0.120 ***	0.006	0.000	−0.071 **	0.445 ***	1												
	7. Knowledge	−0.075 **	0.098 ***	0.071 **	−0.029	0.116 ***	0.127 ***	1											
	8. Negative emotion	0.206 ***	0.020	0.035	−0.037	0.431 ***	0.457 ***	0.249 ***	1										
	9. Trust in government	−0.070 **	−0.005	−0.078 **	−0.025	−0.149 ***	−0.166 ***	0.136 ***	−0.144 ***	1									
Communication	10. Source’s credibility	−0.041	0.117 ***	0.025	0.015	0.091 ***	−0.043	0.070 **	0.012	0.381 ***	1								
	11. Quantity of information	−0.052 *	0.049	−0.074 **	0.019	0.008	−0.146 ***	0.107 ***	−0.106 ***	0.515 ***	0.576 ***	1							
	12. Quality of information	−0.038	0.078 **	−0.060 *	0.007	0.051	−0.096 ***	0.087 ***	−0.045	0.521 ***	0.661 ***	0.866 ***	1						
	13. Receiver’s ability	−0.119 ***	0.001	0.088 ***	0.011	0.137 ***	0.089 ***	0.704 ***	0.184 ***	0.166 ***	0.120 ***	0.156 ***	0.164 ***	1					
Attribution factors	14. Internal attribution	−0.022	−0.048	0.026	−0.036	0.236 ***	0.200 ***	0.097 ***	0.264 ***	−0.217 ***	−0.042	−0.109 ***	−0.084 ***	0.090 ***	1				
	15. External attribution	0.029	−0.234 ***	−0.038	−0.043	0.221 ***	0.172 ***	0.149 ***	0.232 ***	−0.050	−0.015	−0.010	−0.008	0.137 ***	0.471 ***	1			
Response Action	16. Personal/mitigation	0.006	0.090 ***	0.066 **	−0.033	0.231 ***	0.154 ***	0.226 ***	0.205 ***	0.083 ***	0.174 ***	0.156 ***	0.172 ***	0.265 ***	0.079 **	0.043	1		
	17. Personal/adaptation	0.197 ***	0.106 ***	0.090 ***	−0.026	0.416 ***	0.387 ***	0.226 ***	0.471 ***	−0.046	0.174 ***	0.044	0.106 ***	0.242 ***	0.212 ***	0.209 ***	0.435 ***	1	
	18. Public/mitigation	−0.032	0.058*	0.060*	0.019	0.099 ***	0.073 **	0.278 ***	0.123 ***	0.313 ***	0.168 ***	0.235 ***	0.227 ***	0.304 ***	−0.055*	0.034	0.440 ***	0.285 ***	1
	19. Public/adaptation	0.010	0.220 ***	0.032	0.010	0.173 ***	0.127 ***	0.129 ***	0.209 ***	0.163 ***	0.239 ***	0.159 ***	0.197 ***	0.143 ***	0.063 **	−0.035	0.430 ***	0.303 ***	0.367 ***

Note: * *p* < 0.05; ** *p* < 0.01; and *** *p* < 0.001.

**Table 6:** Multiple regression analysis for actions against PM.

		Personal/Mitigation					Personal/Adaptation					Public/Mitigation					Public/Adaptation				
		B	SE	Beta	*t*	Sig.	B	SE	Beta	*t*	Sig.	B	SE	Beta	*t*	Sig.	B	SE	Beta	*t*	Sig.
F1: Demographic factors	Constant	1.013	0.234		4.328	0.000	−0.035	0.205		−0.170	0.865	0.484	0.240		2.018	0.044	0.738	0.227		3.257	0.001
	Gender (male)	−0.009	0.043	−0.006	−0.204	0.839	−0.204 ***	0.038	−0.141	−5.419	0.000	0.015	0.044	0.010	0.341	0.733	0.005	0.042	0.003	0.109	0.913
	Age	0.004 *	0.002	0.073	2.335	0.020	0.006 ***	0.001	0.120	4.481	0.000	0.003 *	0.002	0.061	2.007	0.045	0.009 ***	0.001	0.197	6.416	0.000
	Education level	0.034	0.018	0.058	1.922	0.055	0.049 **	0.015	0.083	3.204	0.001	0.042 *	0.018	0.068	2.335	0.020	0.023	0.017	0.040	1.363	0.173
	Household income	−3.661 × 10^−5^	0.000	−0.039	−1.319	0.188	−9.598 × 10^−6^	0.000	−0.010	−0.395	0.693	2.190 × 10^−5^	0.000	0.022	0.770	0.442	2.133 × 10^−5^	0.000	0.023	0.794	0.427
F2: Risk perception factors	Perceived benefits	0.144 ***	0.035	0.144	4.100	0.000	0.178 ***	0.031	0.174	5.776	0.000	0.063	0.036	0.060	1.751	0.080	0.092	0.034	0.094	2.714	0.007
	Perceived risks	0.043	0.032	0.047	1.352	0.177	0.145 ***	0.028	0.155	5.183	0.000	0.050	0.033	0.052	1.533	0.126	0.038	0.031	0.042	1.222	0.222
	Knowledge	0.032	0.043	0.032	0.748	0.454	−0.001	0.037	−0.001	−0.020	0.984	0.083	0.044	0.078	1.889	0.059	−0.025	0.041	−0.026	−0.613	0.540
	Negative emotion	0.097 *	0.040	0.089	2.451	0.014	0.256 ***	0.035	0.230	7.379	0.000	0.099 *	0.041	0.085	2.426	0.015	0.172 ***	0.038	0.161	4.486	0.000
	Trust in government	0.024	0.032	0.026	0.725	0.468	−0.015	0.028	−0.016	−0.528	0.598	0.247 ***	0.033	0.262	7.427	0.000	0.136 ***	0.031	0.155	4.322	0.000
F3: Communication factors	Source’s credibility	0.052	0.033	0.063	1.590	0.112	0.104 ***	0.029	0.123	3.645	0.000	−0.017	0.034	−0.020	−0.515	0.607	0.103 **	0.032	0.127	3.267	0.001
	Quantity of information	0.072	0.054	0.080	1.334	0.183	−0.035	0.047	−0.038	−0.744	0.457	0.108	0.055	0.113	1.953	0.051	0.003	0.052	0.003	0.051	0.959
	Quality of information	0.018	0.063	0.018	0.283	0.778	0.064	0.055	0.065	1.162	0.245	−0.029	0.064	−0.029	−0.458	0.647	0.019	0.061	0.019	0.305	0.760
	Receiver’s ability	0.171 ***	0.042	0.173	4.127	0.000	0.142 ***	0.036	0.141	3.906	0.000	0.169 ***	0.043	0.162	3.970	0.000	0.068	0.040	0.070	1.700	0.090
F4: Attribution factors	Internal attribution	0.031	0.033	0.032	0.931	0.352	0.036	0.029	0.037	1.261	0.208	−0.067 *	0.034	−0.066	−1.968	0.049	0.071 *	0.032	0.075	2.204	0.028
	External attribution	−0.035	0.031	−0.040	−1.156	0.248	0.073 **	0.027	0.081	2.715	0.007	0.020	0.031	0.021	0.628	0.530	−0.071 *	0.030	−0.083	−2.407	0.016
F-value		12.085 ***					40.178 ***					17.017 ***					14.134 ***				
R^2^/Adjusted R^2^		0.153/0.140					0.375/0.366					0.203/0.191					0.174/0.162				

Note: * *p* < 0.05; ** *p* < 0.01; and *** *p* < 0.001.

**Table 7:** Summary of multiple regression analysis result.

	Personal/Mitigation	Personal/Adaptation	Public/Mitigation	Public/Adaptation
Demographic factors	Age (+)	Gender (Female) (+), Age (+), Education level (+)	Age (+), Education level (+)	Age (+)
Risk perception factors	Perceived benefits (+), Negative emotion (+)	Perceived benefits (+), Perceived risks (+), Negative emotion (+)	Negative emotion (+), Trust in government (+)	Negative emotion (+), Trust in government (+)
Communication factors	Receiver’s ability (+)	Source’s credibility (+), Receiver’s ability (+)	Receiver’s ability (+)	Source’s credibility
Attribution factors	-	External attribution (+)	Internal attribution (−)	Internal attribution (+), External attribution (−)

Note: + sign means the positive impact on response action whereas − sign does the negative influence on it.

## Data Availability

The data presented in this study are available on request from the corresponding author. The data are not publicly available due to regulations and guideline of data.
